# Artocarpin Targets Focal Adhesion Kinase-Dependent Epithelial to Mesenchymal Transition and Suppresses Migratory-Associated Integrins in Lung Cancer Cells

**DOI:** 10.3390/pharmaceutics13040554

**Published:** 2021-04-14

**Authors:** Nongyao Nonpanya, Kittipong Sanookpan, Nicharat Sriratanasak, Chanida Vinayanuwattikun, Duangdao Wichadakul, Boonchoo Sritularak, Pithi Chanvorachote

**Affiliations:** 1 Cell-Based Drug and Health Product Development Research Unit, Faculty of Pharmaceutical Sciences, Chulalongkorn University, Bangkok 10330, Thailand; nonpanya1988@gmail.com (N.N.); 6280112920@student.chula.ac.th (K.S.); toonniich@gmail.com (N.S.); 2 Department of Pharmacology and Physiology, Faculty of Pharmaceutical Sciences, Chulalongkorn University, Bangkok 10330, Thailand; 3 Division of Medical Oncology, Department of Medicine, Faculty of Medicine, Chulalongkorn University, Bangkok 10330, Thailand; Chanida.Vi@chula.ac.th; 4 Department of Computer Engineering, Faculty of Engineering, Chulalongkorn University, Bangkok 10330, Thailand; duangdao.w@chula.ac.th; 5 Department of Pharmacognosy and Pharmaceutical Botany, Faculty of Pharmaceutical Sciences, Chulalongkorn University, Bangkok 10330, Thailand; boonchoo.sr@chula.ac.th

**Keywords:** artocarpin, migration, invasion, epithelial-mesenchymal transition (EMT), cancer stem cells (CSCs), lung cancer

## Abstract

Focal adhesion kinase (FAK) controls several cancer aggressive potentials of cell movement and dissemination. As epithelial–mesenchymal transition (EMT) and the migratory-associated integrins, known influencers of metastasis, have been found to be linked with FAK activity, this study unraveled the potential pharmacological effect of artocarpin in targeting FAK resulting in the suppression of EMT and migratory behaviors of lung cancer cells. Treatment with artocarpin was applied at concentrations of 0–10 μM, and the results showed non-cytotoxicity in lung cancer cell lines (A549 and H460), normal lung (BEAS-2B) cells and primary metastatic lung cancer cells (ELC12, ELC16, and ELC20). We also found that artocarpin (0–10 µM) had no effect on cell viability, proliferation, and migration in BEAS-2B cells. For metastasis-related approaches, artocarpin significantly inhibited cell migration, invasion, and filopodia formation. Artocarpin also dramatically suppressed anchorage-independent growth, cancer stem cell (CSC) spheroid formation, and viability of CSC-rich spheroids. For molecular targets of artocarpin action, computational molecular docking revealed that artocarpin had the best binding affinity of −8.0 kcal/mol with FAK protein. Consistently, FAK-downstream proteins, namely active Akt (phosphorylated Akt), active mTOR (phosphorylated mTOR), and Cdc42, and EMT marker and transcription factor (N-cadherin, Vimentin, and Slug), were found to be significantly depleted in response to artocarpin treatment. Furthermore, we found the decrease of Caveolin-1 (Cav-1) accompanied by the reduction of integrin-αν and integrin-β3. Taken together, these findings support the anti-metastasis potentials of the compound to be further developed for cancer therapy.

## 1. Introduction

Lung cancer is one of the important cancers, with a high rate of mortality, and is characterized by cancer cells growing uncontrollably in the lungs [1]. Lung cancer accounted for approximately 2.1 million cases and approximately 1.8 million deaths in 2018 [2]. Importantly, cancer cells can invade into nearby tissue, and spread through the bloodstream to other organs in the body forming secondary cancers or metastatic cancers [3]. Cancer metastasis has long been recognized as an important cause of the high mortality rate of lung cancer [4]. It is estimated that cancer metastasis is responsible for more than 90% of all cancer-associated deaths [5].

A cellular process called epithelial-to-mesenchymal transition (EMT) is known to facilitate metastasis of cancer cells. EMT was also linked with chemoresistance [6]. EMT is a process in which epithelial cells increase their motility-regulatory signals, resulting in the induction of motile and invasive phenotypes and survival in anchorage-independent conditions [7,8]. The of EMT proteins, such as the E-cadherin and N-cadherin switch, Snail, Slug, TWIST, vimentin, and fibronectin, has been demonstrated to play a key role in cancers [9]. The downregulation of E-cadherin and upregulation of N-cadherin are associated with loss of cell adhesion in cancer cells [10]. Additionally, vimentin is widely expressed in mesenchymal cells and results in EMT, invasion, motility, and tumorigenesis [11]. At focal adhesions, integrins are one of cell adhesion receptors consisting of alpha and beta subunits that facilitate the cell-extracellular matrix (ECM) adhesion, and generate cellular signals resulting in cell survival, migration, and invasion [12].

Likewise, studies have shown that the cellular levels of certain integrins, including αv, α5, β1, and β3, are linked with enhanced cancer migration and metastasis [13]. Additionally, it has been reported that αvβ3 integrin expression is closely associated with cancer invasion [14]. A decrease in integrins β3, β4, and β5 was shown to suppress tumor growth and angiogenesis [15]. Integrin heterodimers, such as αvβ3, αvβ6, α5β1, and α6β4, have also been reported as markers that can differentially affect the adhesion dynamics in cell invasion and migration [16]. Mierke et al. reported that the α5β1 integrin promotes cell invasion via the enhanced transmission and generation of contractile forces [17]. In particular, integrin β1 was shown to trigger protein kinase B (Akt) activation via phosphorylation at serine 473, and integrin β3 was shown to mediate Src activation through Akt and STAT3 signaling [18,19]. In the mechanistic approach, the activation of the pro-survival Akt and focal adhesion kinase (FAK) by directly mediating phosphorylation at Y397 regulates cell migration in various cancers, and they are also involved in the process of integrin engagement [20]. The activation of FAK and its downstream targets, including the Rho family of proteins and Cdc42, are well known to regulate the migration of cancer cells and the formation of filopodia [21].

Several studies have reported that caveolin-1 (Cav-1) plays an important role in cell motility by regulating the polarization of signaling molecules and integrins [22]. In particular, Cav-1 is associated with lung cancer cell migration, invasion, and anoikis resistance [23]. Cav-1 overexpression in cancer has been linked with tumorigenesis and metastasis [24]. A high expression of Cav-1 was observed in metastatic cancer, while a low expression of Cav-1 has been significantly related with overall survival in lung adenocarcinomas [25], thus proposing Cav-1 as a notable therapeutic target for cancer treatment. Studies have suggested the role of Cav-1 in the integrin-mediated ECM remodeling of cancer-associated fibroblasts (CAFs), and in integrin-dependent invasion and cancer metastasis. Cav-1-dependent β1 integrin endocytosis plays a significant role in fibronectin turnover [26]. Cav-1 expression can promote cell migration by regulating cell polarity, which is directly related to actin repolymerization and the integrin switch [27]. Cav-1 was previously shown to control the cellular level of integrins β1, α2, and α5, which are associated with the increased motility and invasion of cancer cells [28]. In addition, the natural compound chrysotobibenzyl was shown to suppress integrins β1, β3, and αv via a Cav-1-dependent mechanism and to inhibit lung cancer cell migration [29].

**Figure 1:**
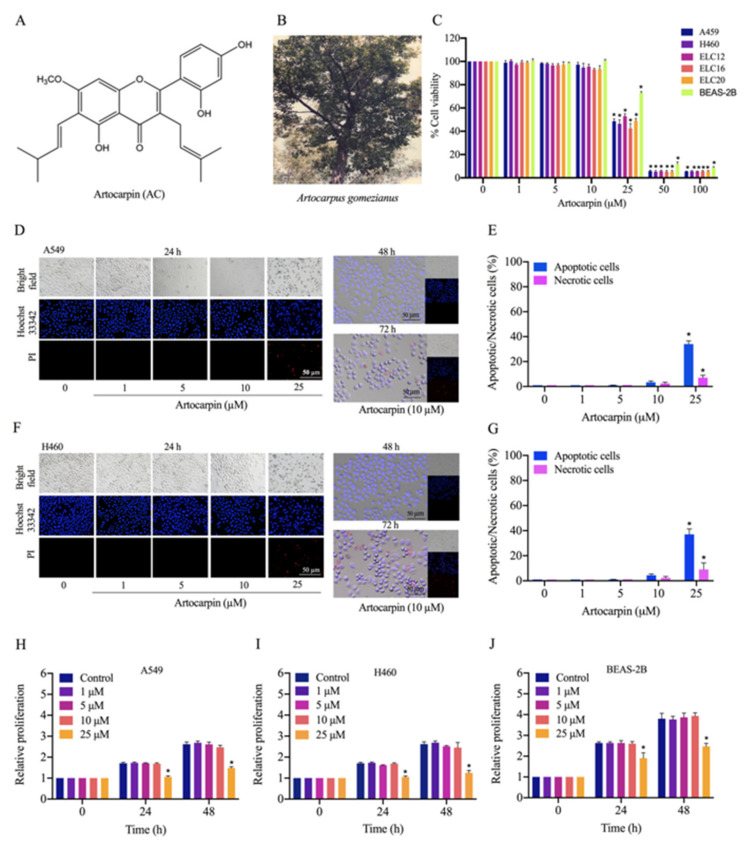
Cytotoxicity of artocarpin on A549, H460, ELC12, ELC16, ELC20, and BEAS-2B cells. (**A**) Chemical structure of artocarpin. (**B**) The whole plant of *A. gomezianus*. (**C**) Cytotoxic effect of artocarpin on A549, H460, ELC12, ELC16, and ELC20 cells. The cells were treated with several doses of artocarpin (0–100 µM) for 24 h; cell viability was measured by MTT assay. The percentage of cell viability was calculated relative to the control group. (**D**–**G**) Apoptotic and necrotic A549 and H460 cells were detected by Hoechst 33342/PI staining and visualized by fluorescence microscopy. Percentage of apoptotic/necrotic nuclei in artocarpin-treated cells was analyzed. (**H**–**J**) The proliferative effect of artocarpin on A549, H460, and BEAS-2B cells was analyzed by MTT assay. Scale bar represents 50 µm. The data are presented as the mean ± SD (*n* = 3). * *p* < 0.05 versus the non-treated control.

Natural products have been shown to have the ability to suppress EMT and metastasis-related integrins [30,31]. Artocarpin, which was first isolated from the root of *Artocarpus heterophyllus*, is one of those. Artocarpin has the molecular formula C_26_H_28_O_6_ and a 5,7,2′,4′-tetrahydroxylated structure with three benzene rings (Figure 1A). Previous studies reported that artocarpin has anti-inflammatory, anti-microbial, anti-oxidative, and anti-cancer activities [32]. However, the effect of artocarpin on lung cancer cells metastasis has not yet been clearly clarified. Consequently, the present study aimed to investigate the effects of artocarpin, a pure compound isolated from *A. gomezianus* (Figure 1B), on the metastasis-related pathways as well as the potential of FAK inhibition in human lung cancer cells.

## 2. Results

### 2.1. Cytotoxicity of Artocarpin on Lung Cancer A549 and H460 Cells

Artocarpin (Figure 1A), isolated from *A. gomezianus*, was used in this study. We first determined the cytotoxicity of this compound on A549, H460, ELC12, ELC16, ELC20, and BEAS-2B cells by the MTT assay. Cells were treated with various concentrations of artocarpin (0–100 μM) for 24 h. The results showed that artocarpin was non-toxic at concentrations less than 25 μM in A549, H460, ELC12, ELC16, ELC20, and BEAS-2B cells (Figure 1C). To investigate the cytotoxic activity of artocarpin in A549 and H460 cells, a nuclear co staining of cells with Hoechst 33342 and propidium iodide (PI) was used for morphological examination. The condensed or fragmented nuclei of apoptotic cells were stained with blue fluorescence of the Hoechst 33342, while the PI-stained cells exhibiting red fluorescence were the necrotic cells. Cells cultivated in the presence of artocarpin at various concentrations (0–25 µM) for 24 h. The compound-treated cells were analyzed for apoptosis and necrosis by Hoechst 33342 and PI. Results showed that artocarpin at 0–10 μM had no significant effect on apoptosis or necrosis, whereas the compound at 25 μM could mediate apoptosis in A549 (Figure 1D,E) and H460 cells (Figure 1F,G). The result showed that artocarpin induced apoptosis at a concentration of 25 µM, while necrosis was rarely detected in all the treated conditions. Furthermore, a cell proliferation assay was performed. Human lung cancer, A549 and H460 cells, and human normal lung BEAS-2B cells were treated with various concentrations of artocarpin (0–25 μM) for 24, and 48 h, and their viability was evaluated by MTT assay. The results indicated that artocarpin at concentration of 1-10 µM had no effect on cell proliferation at 24 and 48 h in all cell types. Meanwhile, artocarpin at 25 μM significantly altered the proliferation of A549 (Figure 1H), H460 (Figure 1I), and BEAS-2B (Figure 1J) cells at 24 and 48 h, respectively. Non-toxic concentrations of artocarpin were used for the further experiments.

### 2.2. Artocarpin Suppresses Cell Migration and Invasion

**Figure 2:**
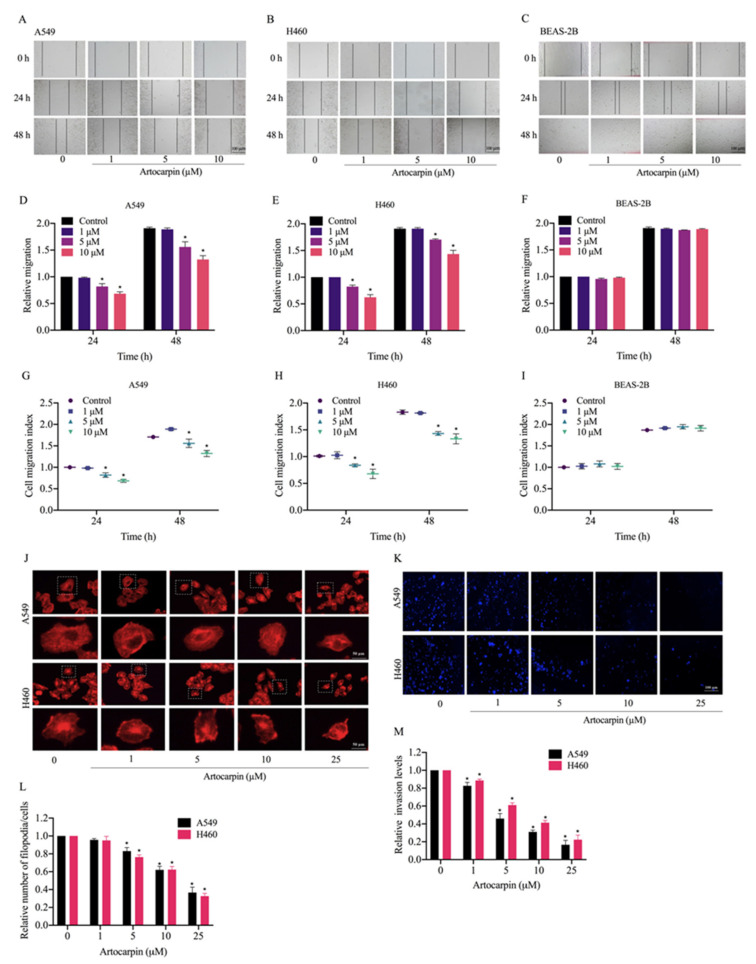
Effect of artocarpin on cancer cell migration, filopodia formation, and invasion. (**A**,**B**) Artocarpin decreased A549 and H460 cell migration but not BEAS-2B cell migration: Cells were treated with non-toxic doses of artocarpin (0–10 μM), and cell migrations at 24 and 48 h were determined. (**C**) Artocarpin at concentrations of 0–10 µM had no effect on cell migration of BEAS-2B cells at 24 h. (**D**–**F**) The relative cell migration of the treated cells was determined, and (**G**–**I**) cell migration index was calculated by normalizing with the proliferation index. (**J**,**L**) Effect of artocarpin on filopodia formation. The artocarpin-treated cells were stained with phalloidin-rhodamine and the filopodia was visualized by fluorescent microscopy. Relative number of filopodia per cell in A549 and H460 cells treated with artocarpin, compared with the control. (**K**,**M**) A549 and H460 cell invasion was examined using transwell invasion assay. The relative cell migration and invasion were calculated by compared to control. After 24 h, the invaded cells were stained with Hoechst 33342 and determined using fluorescence microscopy. Data represent the mean ± SD (*n* = 3). * *p* < 0.05 versus non-treated control.

Cancer metastasis is a multi-step process that includes cell migration and invasion, which are hallmarks of malignant cancer [33,34]. We found that artocarpin was able to significantly attenuate A549 (Figure 2A) and H460 cells (Figure 2B) movement in a dose-dependent manner over the experimental period. Meanwhile, we also found that artocarpin (0–10 µM) had no effect on cell migration in normal lung BEAS-2B cells at 24 h (Figure 2C,F). Artocarpin significantly inhibited A549 cell migration at the concentrations of 5–10 μM at 24 and 48 h, compared with the non-treated control (Figure 2A,D,G). These results were similar to the effects on the migration of H460 cells, whereby H460 cell motility was also significantly suppressed by 5–10 μM artocarpin at 24 and 48 h (Figure 2B,E,H). Consistently, the effect of the compound on filopodia formation was evaluated and the results indicated that artocarpin at non-cytotoxic doses suppressed the number of filopodia in these lung cancer cells (Figure 2G,L). In addition, cell invasion was detected using a transwell Boyden chamber assay, whereby the cells were treated with artocarpin (0–25 μM) for 24 h. We found that artocarpin was able to decrease the number of migrating and invading cells through the matrigel layer of the assay at 24 h in a dose-dependent manner, compared with the non-treated control (Figure 2K,M). An invasion assay was also performed, and it was found that artocarpin could decrease the number of invaded A549 and H460 cells.

### 2.3. Artocarpin Suppresses the Anchorage-Independent Growth and CSC-Like Phenotype of Lung Cancer Cells

**Figure 3:**
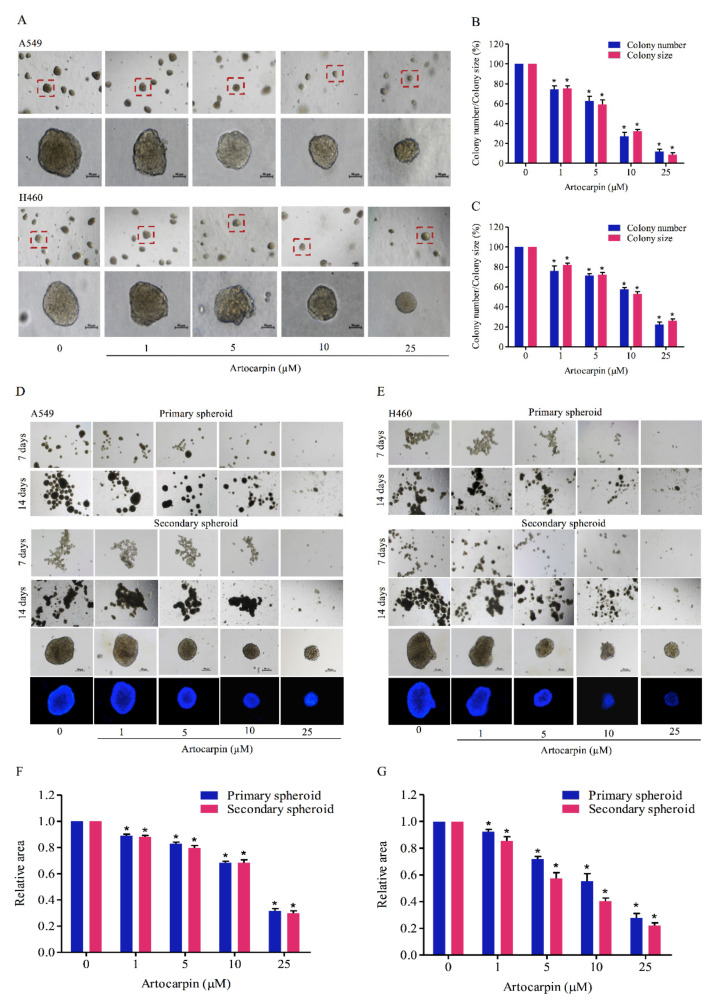
Artocarpin suppresses anchorage-independent growth and cancer stem cell (CSC)-like phenotypes of lung cancer cells. (**A**–**C**) A549 and H460 cells were pre-treated with non-toxic doses of artocarpin (0–10 μM) and a toxic dose (25 μM) at 37 °C for 24 h, and were assigned to an anchorage-independent growth assay for 2 weeks. (**D**–**G**) Cells were treated with artocarpin (0–25 μM) for 24 h, and the cells were suspended and allowed to form primary spheroids for 14 days. After dissociation of the primary spheroids, the cells were allowed to form secondary spheroids. Next, the spheroid of cancer stem cell (CSC)-rich population was investigated. All data are represented as mean ± SD (*n* = 3). * *p* < 0.05, versus the non-treated control.

The sustained viability and growth of lung cancer cells in an anchorage-independent condition have been suggested to link with potentials of the cancer cells to metastasis [8,35]. We next determined the effect of artocarpin in attenuating cell growth and survival under detachment conditions. Cells were grown in soft agar in the presence or absence of artocarpin for 14 days. The size and number of the colonies formed were determined. As illustrated in Figure 3A,B, the number of colonies on A549 cells were significantly reduced by artocarpin treatment at 1, 5, 10, and 25 μM by 75.6%, 45.4%, 33.2%, and 11.3%, respectively, and the percentage colony sizes in response to artocarpin at concentrations of 1, 5, 10, and 25 μM were 69.0%, 47.6%, 34.3%, and 15.3%, respectively. Additionally, the results indicated that the number of colonies on H460 (Figure 3A,C) cells were significantly reduced by artocarpin at 1, 5, 10, and 25 μM by 78.0%, 71.3%, 57.6%, and 28.3%, respectively, and the percentage colony sizes in response to the compound at 1, 5, 10, and 25 μM were 81.6%, 73.3%, 56.6%, and 31.3%, respectively. These results revealed that artocarpin at non-toxic concentrations could suppress the survival and growth of the lung cancer cells in the detached condition.

Having shown the effect of artocarpin on the anchorage-independent growth of cancer cells, we next evaluated the activity of artocarpin to suppress the CSC-like phenotypes. A549 and H460 cells were treated with non-cytotoxic concentrations (0–10 µM) and a toxic dose (25 µM) of artocarpin for 24 h, and the cells were subjected to the spheroid-formation assay. The primary and secondary spheroids were formed as described in Materials and Methods, and we found that the control cells had the ability to form dense tumor spheroids, whereas cells treated with artocarpin exhibited reduced number of tumor spheroids (Figure 3D,E), suggesting that artocarpin had a suppressing effect on the CSC populations in these cells. Artocarpin at concentrations of 1–10 µM, which was considerately non-cytotoxic, was able to significantly decrease the number and size of the primary and secondary spheroids compared with the control in lung cancer A549 (Figure 3D,F) and H460 cells (Figure 3E,G).

**Figure 4:**
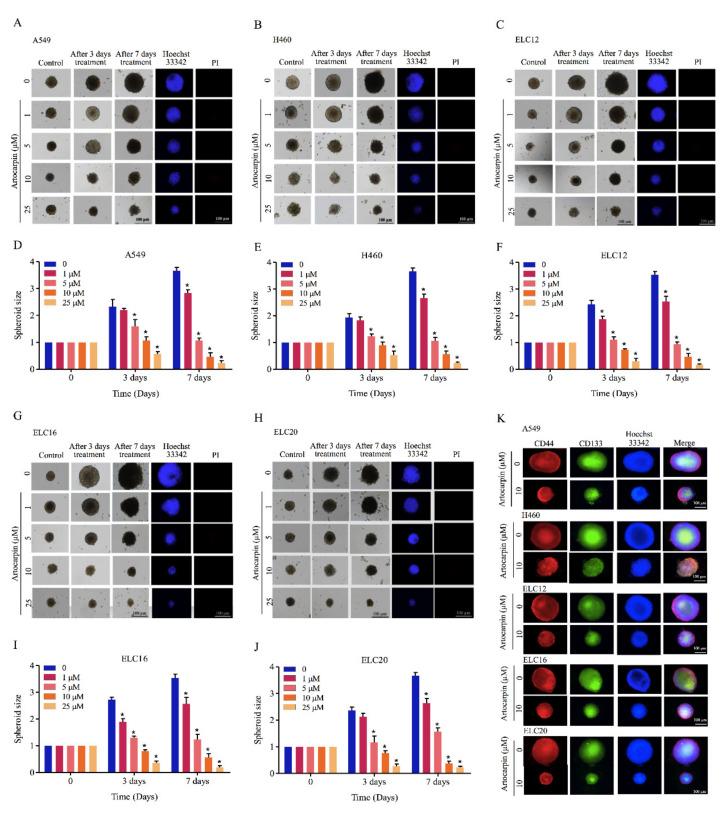
Artocarpin suppresses CSC growth in a CSC-rich population. (**A**,**D**) A549, (**B**,**E**) H460, (**C**,**F**) ELC12, (**G**,**I**) ELC16, and (**H**,**J**) ELC20 secondary spheroids were selected and treated with artocarpin (0–10 µM) for 3 and 7 days. Phase-contrast images of secondary spheroids at days 0, 3, and 7 and untreated cells were determined. At 7 days, single spheroid was stained with Hoechst 33342 to aid visualization of the cell nucleus. (**K**) The expression levels of CD44 and CD133 were determined using the anti-CD44 and anti-CD133 antibody, followed by the Alexa Fluor 594-labeled secondary antibody, and Alexa Fluor 488-labeled secondary antibody and determined using fluorescence microscopy. Data represent the mean ± SD (*n* = 3). * *p* < 0.05, versus non-treated control.

Additionally, CSC-rich populations of lung cancer cells were established. Secondary spheroids were prepared in a 24-well ultralow-attachment plates for 14 days. The single spheroid containing CSC-rich cancer cells was isolated and grown in the 96-well plate. The CSC spheroids were treated with artocarpin (0–25 μM) for 7 days. Figure 4 shows the representative images of the CSC-rich spheroids in the control and artocarpin-treated cells at days 0, 3, and 7. Treatment of the CSC-rich populations with 0-25 µM artocarpin caused the reduction of CSC survival in both cells, with a significant decrease in the size of the A549 CSC spheres by approximately 40%, 63%, and 81% at day 3 after treatment with 5, 10, and 25 µM artocarpin, respectively, compared with the control (Figure 4A,D). The CSC survival was found to be dramatically reduced as indicated by the condensed and fragmented nuclei stained with DNA dye Hoechst 33340. At day 7, results showed a further decrease of size of A549 spheroids by approximately 40%, 65%, 89%, and 95% relative to the control in response to 1, 5, 10, and 25 µM artocarpin, respectively. For the H460 cells, in which the treatment of H460 CSC spheres with artocarpin caused shrinkage of the spheroids (Figure 4B,E). The suppression of artocarpin was first detected at the dose of 5 µM at day 3 (approximately 45% size reduction of the CSC spheres); the size reduction was about 45%, 65%, 87%, and 95% relative to the non-treated cells in response to 1, 5, 10, and 25 µM artocarpin at day 7, respectively. Similarly, treatment of the CSC spheres of primary lung cancer cells with artocarpin at 0–25 µM significantly resulted in the dramatic reduction of CSC survival and spheroid size of ELC12 (Figure 4C,F), ELC16 (Figure 4G,I), and ELC20 cells (Figure 4H,J). Moreover, artocarpin at dose of 10 µM significantly decreased the number of organoids, buds, and area compared with the control in A549, H460, ELC12, ELC16, and ELC20 cells (Figure S1). Taken together, artocarpin exhibited anti-metastasis activities as it could suppress growth under anchorage-independent conditions, cancer cell migration, invasion, and could also suppress the CSC-like phenotypes.

### 2.4. Artocarpin Attenuates EMT via the Suppression of the FAK/Akt/mTOR Signaling Pathway

**Figure 5:**
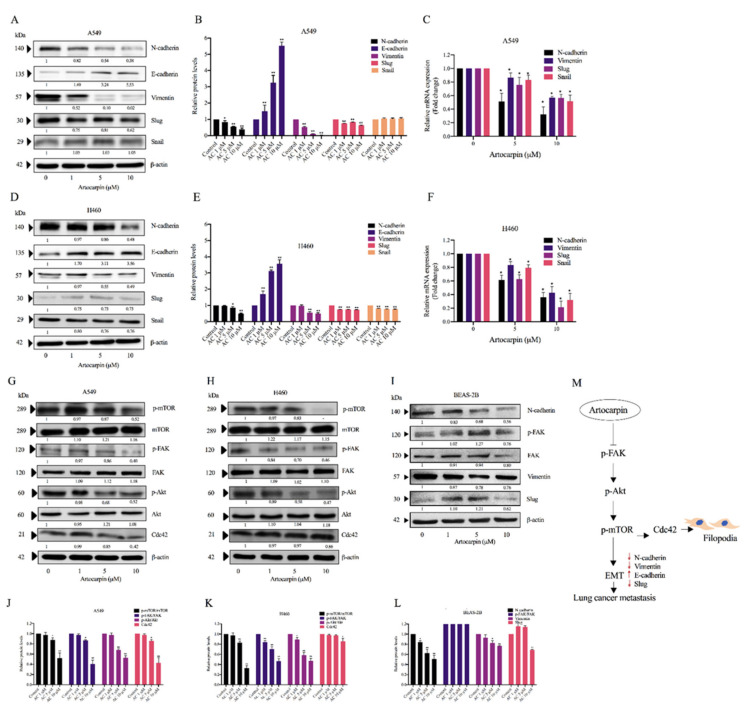
Effect of artocarpin on epithelial to mesenchymal transition (EMT): A549, H460, and BEAS-2B cells were treated with various concentrations of artocarpin (0–10 μM) for 24 h. (**A**–**I**) The expression levels of EMT protein markers were investigated by Western blotting, as well as the mRNA expression levels of EMT markers (**C**,**F**) were obtained by quantitative real-time polymerase chain reaction. Blotting membranes were probed with β-actin to confirm equal loading of samples. (**G**–**I**) The expression levels of Cdc42, mTOR, FAK and Akt proteins, and their phosphorylated forms, were investigated. (**J**–**L**) Relative protein levels were quantified using densitometry. β-actin was used as loading control. The data are shown as mean ± SD (*n* = 3). * *p* < 0.05, ** *p* < 0.01 versus non-treated control. (**M**) Schematic mechanism of artocarpin in suppression of EMT markers and filopodia formation in lung cancer cells.

EMT has also been linked to the induction of cancer metastasis. The effect of artocarpin on EMT was determined by Western blotting. A549 and H460 cells were treated with various non-toxic concentrations of artocarpin for 24 h. The EMT markers, namely N-cadherin, E-cadherin, vimentin, Slug, and Snail, were determined and the results revealed that treatment of the cells with the compound could suppress EMT markers compared with the control cells. In the treated cells, it was found that artocarpin significantly suppressed the levels of the EMT markers, such as N-cadherin, vimentin, and Slug, in A549 (Figure 5A,B) and H460 (Figure 5D,E) cells. Similar results were found for the A549 and H460 cells, in which treatment with artocarpin resulted in a dramatic decrease the mRNA levels of N-cadherin, Vimentin, Slug, and Snail in a dose-dependent manner (Figure 5C,F). Moreover, the upstream regulatory signals of EMT and the controllers of cell migration, such as p-FAK, p-Akt, and p-mTOR were determined. The results demonstrated that artocarpin decreased the levels of p-FAK, p-Akt, and p-mTOR in A549 and H460 cells. Artocarpin was able to decrease the active form of mTOR (phosphorylated at Ser2448) in tested lung cancer cells at the concentrations of 5-10 µM (*p* < 0.05). In addition, the active FAK (phosphorylated at Tyr397, p-FAK) was found to be suppressed by the treatment with 5–10 µM artocarpin in A549 cells (Figure 5G,J); however, we found a significant reduction of the p-Akt protein only at concentrations of 1–10 µM in H460 cells (Figure 5H,K). Additionally, the active form of Akt, i.e., p-Akt (phosphorylated at Ser473), was also found to be attenuated in response to 5–10 µM and 1–10 µM artocarpin in A549 and H460 cells, respectively. Several studies have reported that Cdc42 plays a role in the formation of filopodia [36]. Therefore, the expression of Cdc42 protein was determined in artocarpin-treated cells. We found that artocarpin-treated cells showed a significant reduction in the level of Cdc42 (Figure 5G,H). In normal lung epithelial cells, artocarpin could decrease the levels of the EMT markers as N-cadherin, Vimentin, and Slug in BEAS-2B cells; however, the compound showed no effect on FAK activation (Figure 5I,L). Figure 5M shows a schematic mechanism for artocarpin activity in the suppression of EMT and cell migration. Immunofluorescence staining of p-FAK further confirmed the attenuation effect of artocarpin on FAK-dependent pathway, as we found a significant reduction of p-FAK protein signal in response to 5 and 10 µM in both A549 and H460 cells (Figure S2). These results suggest that artocarpin suppressed EMT as well as inhibited human lung cancer cell motility through the inhibition of the FAK/Akt/mTOR signaling pathway.

### 2.5. Molecular Docking Simulation Reveals the Artocarpin Interaction with the FAK Protein

**Figure 6:**
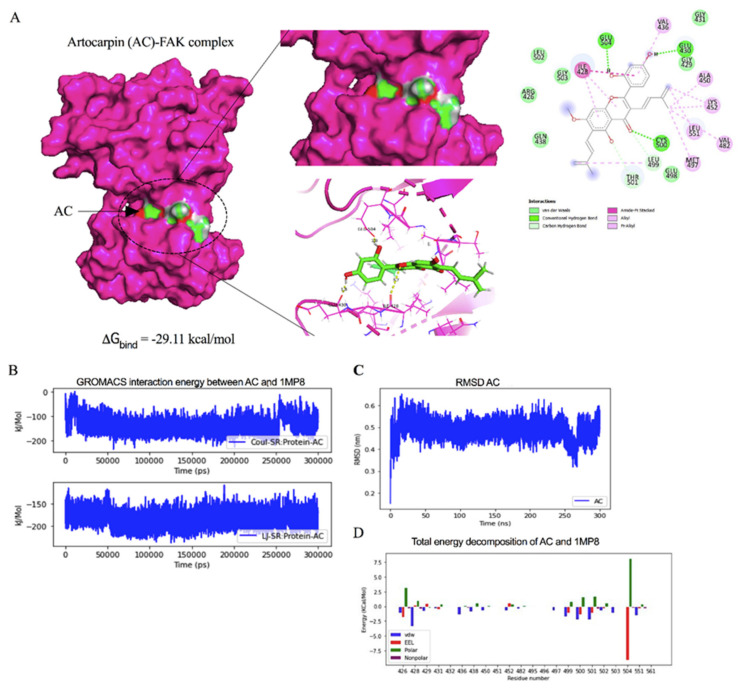
Docked model depicting interaction of artocarpin (AC) with FAK protein. (**A**) Binding mode and docking energy of AC bound to the binding site of FAK taken from the MD study. (**B**) The GROMACs interaction energy between artocarpin (AC) and FAK (1MP8). (**C**) The RMSD plot for interaction of artocarpin-FAK complex during 300 ns of molecular dynamic simulation. (**D**) Total energy decomposition of artocarpin-FAK complex using the gmx_MMPBSA program.

Having shown that artocarpin had an attenuating effect on FAK function, we next tested for the possibility of the compound and FAK protein interaction and found that the binding affinity score calculated from Auto Dock vina was -8.0 kcal/mol. The binding affinity of the artocarpin-FAK complex was determined. The Molecular Mechanics/Generalized Born Surface Area (MM/GBSA) method was applied on the last 200-ns of simulations. The total contributing amino acids of artocarpin-FAK complex is shown in Figure 6A and Video S1, the key binding residues ILE428, GLU430, CYS500, and GLU504 involved in artocarpin binding were identified to be those of the FAK inhibitors. Importantly, the ligand binding mode of FAK models shared a structurally related characteristic, in which the aromatic moiety of artocarpin approached the key residue ILE428. Ligand positional RMSD was generated to determine the binding stability of artocarpin-FAK complex. The computed RMSD values of the ligand artocarpin in complex with FAK was indicated high stability (Figure 6C). The residue decomposition free energy calculation based on the MM/GBSA method was also used to detect key amino acid residues involved in ligand binding of artocarpin within the ATP-binding pocket of FAK protein. Binding free energy (ΔG_bind_) and its energy components (kcal/mol) are summarized in Table 1. The ∆G_bind_ of artocarpin-FAK complex was −29.11 kcal/mol. Due to the structure of artocarpin (Figure 6D), the molecular mechanics energy (∆E_MM_) revealed that van der Waals interaction (∆E_vdW_) was the main force driving artocarpin/FAK complexation (∆E_vdW_ of −43.15 kcal/mol) for artocarpin/FAK complex.

### 2.6. Artocarpin Mediates Integrin Switch via a Cav-1-Dependent Pathway

**Figure 7:**
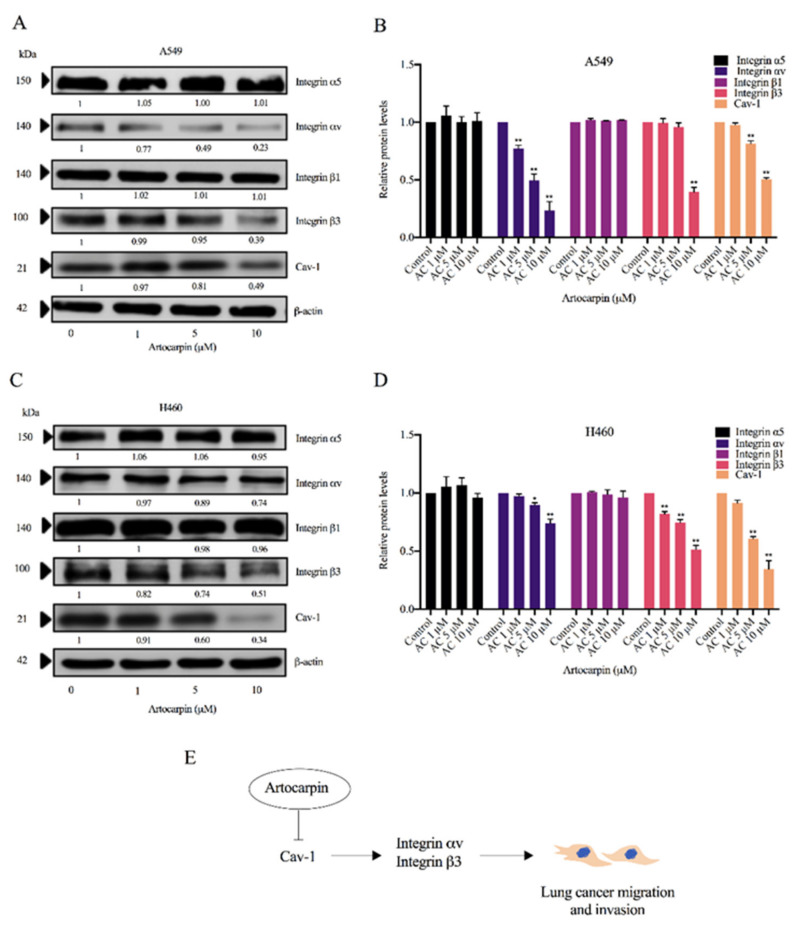
Effect of artocarpin on integrin expression. (**A**) A549 and (**C**) H460 cells were treated with artocarpin (0–10 μM) for 24 h. The protein expression levels of integrins β1, β3, αν, α5, and Cav-1 were investigated by Western blotting. (**B**,**D**) Relative protein expression levels were quantified using densitometry. Blotting membranes were probed with β-actin to confirm the equal loading of the samples. Data represent the mean ± SD (*n* = 3). * *p*< 0.05, ** *p* < 0.01 versus non-treated control. (**E**) Schematic mechanism of artocarpin-mediated integrin switch in human lung cancer A549 and H460 cells.

The scaffolding protein Cav-1 was shown to have an impact on behaviors of cancer cells, including cell movement and anoikis resistance. In addition, Cav-1 was shown to control the expression pattern of integrins. As certain integrins including integrins αv and β3 were shown to associate with progression and anchorage-independent growth of cancers, we investigated the effect of artocarpin on altering the pattern of the integrins as well as the Cav-1 expressions. The cells were similarly treated with non-toxic concentrations of artocarpin (0–10 μM) for 24 h. The protein expression levels of integrins α5, αv, β1, and β3 as well as Cav-1 were determined by Western blotting. We found that artocarpin significantly decreased the protein expression of integrins αv and β3 as well as Cav-1 in both A549 (Figure 7A,B) and H460 (Figure 7C,D) cells when compared with the control. Figure 7E shows a schematic mechanism for the artocarpin-mediated integrin switch in human lung cancer cells. Our previous study indicated that Cav-1 plays a key role in the regulation of cancer metastasis [23]. The Cav-1 protein has been demonstrated to control anoikis resistance in lung cancer cells [37]. Thus, we suggest that Cav-1 may inhibit cell migration via integrins αv and β3.

## 3. Discussion

The majority of cancer deaths are associated with cancer metastasis [3]. Despite new cancer therapy and novel strategic options continually being developed, the overall survival and cancer-free periods of malignant cancer patients are still low. Therefore, the development of therapies that inhibit the metastatic spread and growth of cancer cells are urgently needed to improve clinical outcomes.

It was shown that mesenchymal phenotypes of cancer cells render augmented metastasis potentials, such as cell migration, invasion, and anoikis resistance [38]. The EMT process helps change the adherent epithelial phenotypes of the cancer cells to more motile with increased invasive abilities. The indicators of EMT are the upregulation of N-cadherin followed by the downregulation of E-cadherin, and the upregulation of Slug, Snail, and Vimentin [10]. Importantly, E-cadherin is an adherent protein, and the loss of its expression enhances cell detachment, which is an early step of metastasis [39]. In contrast, N-cadherin increases cancer cell motility [40]. Artocarpin, a natural compound isolated from *A. heterophyllus*, has previously been shown to exhibit several pharmacological properties, including anti-inflammatory, anti-microbial, anti-oxidative, and anti- cancer [32,41]. Herein, we revealed novel information about the active artocarpin compound on EMT. Treatment of lung cancer cells with artocarpin at non-toxic concentrations significantly reduced the expression levels of EMT markers, such as N-cadherin, Vimentin, and Slug, while increasing E-cadherin (Figure 5), indicating the effect of artocarpin in terms of its inhibition of EMT.

Furthermore, artocarpin was demonstrated to reduce the cellular levels of integrins αv and β3 (Figure 7), which have previously been reported to have metastasis-potentiating activities [42]. A previous study showed that a high level of integrin αv promotes brain metastasis [43]. In addition, integrins αv and β3 were reported to facilitate cancer progression and the anchorage-independent growth of cancer cells [44]. In terms of their mechanism of action, a study on prostate cancer revealed that integrins αv and β3 promote cancer cell migration by activating the FAK-dependent mechanism [45]. The regulatory role of such integrins on FAK was also found in the present study. We found that in response to artocarpin treatment, the activated FAK was attenuated concomitantly with the depletion of integrins αv and β3 (Figure 5). Importantly, FAK is a protein tyrosine kinase that regulates cancer cell adhesion, migration, and metastasis by activating signal transducers, including PI3K, Akt, mTOR, and RAS [46,47,48,49]. Several small molecule inhibitors of FAK that target the ATP-binding site have been reported, such as BI853520 [50], PF 573,228 Pfizer [51], and NVP-TAE226 [52]. In this study, we used molecular docking to explore the characteristic of interaction between artocarpin and FAK protein at ATP-binding pocket and found that artocarpin has multiple interactions with surrounding residues, including ILE428, GLU430, CYS500, and GLU504 (Figure 6A). Previously, ILE428, VAL436, ALA452, VAL484, LEU501, GLU505, GLU506, LEU553, GLY563, LEU567, and SER568 are reported as crucial residues [53]. Thus, the data of targeting the ATP-binding site of FAK by artocarpin can be used for the development of this compound for cancer therapy. Various pieces of evidence have revealed the role of Cav-1 in the regulation of cancer metastasis. Cav-1 was shown to be highly expressed in many tumors and such a high protein level was correlated with cancer invasion, metastasis, and poor survival [54]. In addition, Cav-1 was demonstrated to positively regulate anoikis resistance in lung cancer cells [23]. It was previously suggested that Cav-1 is involved in the integrin switch [55]. Furthermore, chrysotobibenzyl was shown to suppress cancer cell migration via a Cav-1-dependent integrin switch [28]. As our results also showed a decrease in Cav-1 as well as integrins αv and β3 (Figure 7), it is possible that artocarpin may at least in part inhibit cancer cell motility via a Cav-1-dependent downregulation of such integrins.

**Figure 8:**
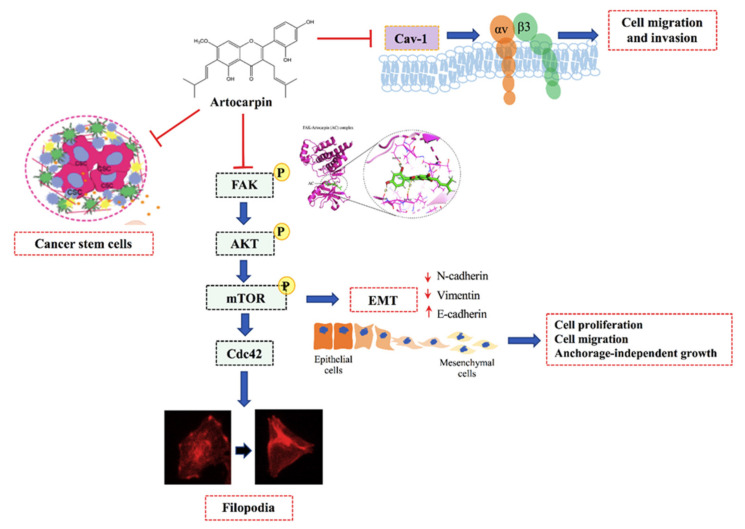
Schematic mechanism of artocarpin suppressing EMT and CSC in lung cancer cells.

Regarding EMT, it was shown that the Akt and mTOR pathways control the EMT process [56]. Irie et al. reported that Akt has a function in regulating growth factor-stimulated EMT and cell migration [57]. Recent studies have shown that FAK has been identified as a critical regulator of cancer cell survival, proliferation, and migration [58,59]. The inhibition of this FAK molecule may benefit cancer therapy in several approaches. It was shown that signals from microenvironment of cancer can activate several survival pathways including Akt and NF-κB. Interestingly, the blockage of FAK was shown to suppress such survival signals from stromal of cancer cells, inhibit invasion, and induce cell apoptosis [60,61]. Likewise, as mTOR is a downstream target of Akt, Gulhati et al. demonstrated that mTORC1 and mTORC2 are the important regulators of EMT [62]. Lamanuzzi et al. reported that mTORC2 regulates tumor angiogenesis in multiple myeloma (MM), and suggested that the combined mTOR inhibitor PP242 with lenalidomide and bortezomib exhibited synergistic activities in inhibiting angiogenesis in vivo in the Chick Chorioallantoic Membrane (CAM) and Matrigel^®®^ plug assays [63]. Consistent with the above findings, we found that artocarpin reduced the active Akt and mTOR, followed by a reduction of the EMT markers, revealing the molecular cascade of action of the compound in the regulation of EMT (Figure 5). Accordingly, FAK and mTOR also play an essential role in the regulation of cancer cell migration, invasion and metastasis as well as EMT process [64]. Zheng et al. suggested that the FAK/PI3K/Akt signaling pathway mediate EMT of endometrial cells in adenomyosis following the upregulation of FAK [64]. On the other hand, some studies have suggested that mTOR complexes regulate EMT via RhoA and Rac1 signaling pathways in prostate cancer [65]. Indeed, the regulation of endothelial cell (EC) functions that are tightly regulated by anti-angiogenic factors via the suppression of the αvβ3/FAK/mTOR signaling pathway [66]. In summary, we have revealed the novel effects of artocarpin in suppression of cell migration, EMT, CSC-like phenotypes, and the migratory-associated integrins via the interaction with FAK resulted in the suppression of the FAK/Akt/mTOR pathway (Figure 8). These findings demonstrate a promising distinctive effect of artocarpin on lung cancer cells that may lead to opportunities to use as an anti-cancer compound in cancer therapy.

## 4. Materials and Methods

### 4.1. Artocarpin Isolation

Artocarpin was isolated from the heartwood of *A. gomezianus* [67]. The dried and powdered heartwood (3.8 kg) was extracted with MeOH (12 L) (Merck, Darmstadt, Germany) to afford the crude MeOH extract (160 g) after removal of the solvent. This crude MeOH extract was subjected to vacuum-liquid chromatography (Waters, Milford, MA, USA) on silica gel (EtOAc and hexane, gradient) to give five fractions (A–E). Fraction C (1.3 g) was fractionated by column chromatography over silica gel eluting with a EtOAc (Merck, Darmstadt, Germany) and hexane (Merck, Darmstadt, Germany) (gradient) and then purified by Sephadex LH-20 (MeOH) (Amersham Pharmacia Biotech AB, Uppsala, Sweden) to give artocarpin (62 mg). The artocarpin purity was determined by NMR spectroscopy (Bruker, Massachusetts, USA). Artocarpin with more than 95% purity was diluted with complete culture medium to achieve the desired concentrations. The chemical structure of artocarpin shown in Figure 1A.

### 4.2. Patient-Derived Primary Lung Cancer Cell Line Preparation from Malignant Pleural Effusion

The primary lung cancer cells were prepared from patient-derived pleural effusions of advanced stage or recurrent lung cancer (non-small cell lung cancer) patients at the King Chulalongkorn Memorial Hospital. The procedure and protocol were approved by the Ethics Committee of the Faculty of Medicine, Chulalongkorn University, Bangkok, Thailand (IRB 365/62). This work was performed in accordance with the principles of World Medical Association Declaration of Helsinki. The cells were prepared from approximately 500–1000 mL pleural effusion through thoracentesis. These cells were cultivated in RPMI medium smented with 10% FBS, 2 mM L-glutamine, and 100 units/mL of each of penicillin and streptomycin.

### 4.3. Cell Cultures and Reagents

A549 and H460 cells were purchased from the American Type Culture Collection (Manassas, VA, USA). A549 cells were cultured in Dulbecco’s Modified Eagle’s Medium (DMEM) (Gibco, Grand Island, NY, USA), and H460 cells in Roswell Park Memorial Institute (RPMI) 1640 medium (Gibco, Grand Island, NY, USA), both incubated at 37 °C with 5% CO_2_. Patient-derived primary cells (ELC12, ELC16, and ELC20) were cultured in RPMI 1640 medium [68]. Meanwhile, normal (non-cancerous) lung (BEAS-2B) cells was maintained in DMEM medium (Gibco, Grand Island, NY, USA). The culture media were smented with 10% (*v*/*v*) fetal bovine serum (FBS) (Merck, DA, Germany), 100 U/mL of penicillin and 100 µg/mL streptomycin (Gibco, Grand Island, NY, USA), and 2 mM L-glutamine (Gibco, Grand Island, NY, USA). The cells were cultured at 37 °C with 5% CO_2_ in a humidified incubator. 3-(4,5-Dimethylthiazol-2-yl)-2, 5-diphenyltetrazoliumbromide (MTT), dimethyl sulfoxide (DMSO), Hoechst 33342, propidium iodide (PI), Phalloidin-Rhodamine, and bovine serum albumin (BSA) were purchased from Invitrogen (Eugene, USA). Antibodies directed against Slug (#9585), Snail (#3879), Vimentin (#5741), N-cadherin (#13116), E-cadherin (#3195), FAK (#3285), p-FAK (#3283), Akt (#4685), p-Akt (#4060), mTOR (#2983), p-mTOR (#5536), Cav-1 (#3267), Cdc42 (#2466), β-actin (#4970), Integrin αv (#4600), Integrin α5 (#4705), Integrin β1 (#4706), Integrin β3 (#4702), Integrin β5 (#4708) as well as the respective secondary antibodies were purchased from Cell Signaling Technology (Danvers, MA, USA).

### 4.4. Cell Viability Assay and Cell Proliferation Assay

Monolayers of the cells at a density of 1 × 10^4^ cells/well were seeded onto 96-well plates. Cells were cultured in the presence of various concentrations of artocarpin (0–100 µM). Cell viability was determined by incubating the cells with 400 μg/mL of MTT for 4 h at 37 °C. The formazan crystals were solubilized in DMSO and the formed color was measured at wavelength 570 nm with microplate reader (Anthros, Durham, NC, USA). The percentage of viable cells was calculated in relation to the non-treated control cells.

### 4.5. Nuclear Staining Assay

Nuclear co-staining with Hoechst 33342 (Sigma Chemical, St. Louis, MO, USA) and propidium iodide (PI) (Sigma Chemical, St. Louis, MO, USA) were used to detect the level of apoptotic and necrotic cells. A549 and H460 cells were seeded at a density of 1 × 10^4^ cells/well of 96-well plates for overnight and treated with artocarpin at various concentrations (0–25 µM) for 24 h. After 24 h, the treated cells were co-stained with 10 μM of Hoechst 33342 and 5 μM of propidium iodide (Sigma, St. Louis, MO, USA) at 37 °C for 10 min. Cells were visualized and imaged by fluorescence microscopy (Olympus DP70, Melville, NY, USA) and then the percentages of apoptotic and necrotic cells were determined.

### 4.6. Anchorage-Independent Growth Assay

Lung cancer A549, and H460 cells were pre-treated with non-cytotoxic concentration of artocarpin (0–10 µM) and toxic dose (25 µM) for 24 h and assigned to anchorage-independent growth assay. Briefly, the bottom layer was prepared by using a 1:1 mixture of complete culture medium containing 10% FBS (Merck, DA, Germany) and 1% agarose gel. After solidification, 1 x 10^3^ cells were suspended in 0.3% agarose gel in complete medium and seeded onto the bottom layer. The culture medium smented with 10% FBS (Merck, DA, Germany) was added over the upper layer. Cells were allowed to form colonies and grow for 2 weeks, and the complete medium was added every 2 days to prevent dryness. The colony was counted and captured using a phase-contrast microscope (Nikon ECLIPSE Ts2, Tokyo, Japan). Colony number and size were determined and compared to those of the control cells.

### 4.7. Spheroid Formation Assay

A549, H460, ELC12, ELC16, and ELC20 cells were pretreated with non-cytotoxic concentration of artocarpin (0–10 µM) and toxic dose (25 µM) for 24 h and assigned to spheroids using a previously described method [69]. The treated cells at a density of 3 × 10^3^ cells/well were cultured in an ultralow-attachment plate. The culture medium containing 1% FBS (Merck, DA, Germany) was added every 2 days and the cells were grown for 7 days to form primary spheroids. The primary spheroids were dissociated and suspended to single cells. Cells were allowed to form secondary spheroids in a 24-well ultralow-attachment plate for 21 days. After 14 and 21 days, the numbers and sizes of spheroids were detected and captured using a phase-contrast microscope (Nikon ECLIPSE Ts2, Tokyo, Japan).

For single three-dimensional (3D) spheroid-formation assay, the method was modified from a previously described method [69]. ELC12, ELC16, ELC20, A549, and H460 cells were cultured in a 24-well ultralow-attachment plates at the density of 3 × 10^3^ cells/well, and allowed to form for 7 days. After incubation, primary spheroids were resuspended into single cells, and seeded onto a 24-well ultralow-attachment plates for 14 days to form secondary spheroids. After 14 days, secondary spheroids were detected and collected. Single spheroid was transferred at one spheroid per well to a 96-well ultralow-attachment plates and treated with artocarpin (0–25 µM) at 37 °C for 7 days. After 7 days, Single spheroid was co-stained with Hoechst 33342 (Sigma, St. Louis, MO, USA) and PI (Sigma Chemical, St. Louis, MO, USA) at 37 °C for 10 min. The single spheroid was determined and captured randomly using a fluorescence microscope (Nikon ECLIPSE Ts2, Tokyo, Japan).

### 4.8. Migration and Invasion Assay

Wound healing and transwell migration assay were used for detecting cell migration. For the wound-healing assay, cells at the density of 2 × 10^4^ cells were seeded in 96-well plates. After the complete monolayer of the cells was formed, wound scratches were generated using a P200 micropipette tip, and detached cells were removed by washing with 1 × PBS (Gibco, Grand Island, NY, USA). Monolayers cells were incubated with a non-toxic concentration of artocarpin (0–10 μM) and a toxic dose (25 µM) at 37 °C for 24 and 48 h. Images were determined at indicated time points, the pictures of cell migration were measured and captured by a fluorescence microscope (Nikon ECLIPSE Ts2, Tokyo, Japan). The wound area was quantified by Image J software (NIH, Bethesda, MD, USA). The percentage of the altered space of wound area was calculated as follows: Change in the wound space (%) = (average space at time (0–24 h, 48 h)/ average space at time 0 h) × 100. Moreover, the relative cell migration of the treated cells was determined, and cell migration index was analyzed by normalizing with the proliferation index.

Additionally, cell invasion assay was performed using a transwell Boyden chamber (8 μm pore size; BD Bioscience, MA, USA). The upper chamber was prepared and coated with 0.5% Matrigel (BD Biosciences, San Jose, CA, USA) at 37 °C for overnight. A549 and H460 cells were treated with artocarpin at non-toxic dose (0–10 µM) and toxic dose (25 µM) at 37 °C for 24 h. After treatment, 2 × 10^4^ cells in a serum-free medium added into the upper chamber with a 0.8 μm pore-sized membrane, while the complete medium containing 10% FBS (Merck, DA, Germany) was added into the lower chamber, and incubated at 37 °C for 24 h. The non-invading cells in the upper chamber were swabbed out and those on the lower chamber of the membrane were fixed with cold methanol (Merck, DA, Germany) for 10 min followed by incubation with Hoechst 33342 (Sigma, St. Louis, MO, USA) for 10 min. Next, migrating cells were captured randomly using a fluorescence microscope (Nikon ECLIPSE Ts2, Tokyo, Japan).

### 4.9. Cell Morphology and Filopodia Characterization

A phalloidin-rhodamine staining was performed to analyze the effect of active compound on cell morphology and filopodia characterization as previously described [29]. Artocarpin-treated A549 and H460 cells were fixed with 4% paraformaldehyde (Sigma Chemical, St. Louis, MO, USA) at 37 °C for 15 min. After fixation, the treated cells were rinsed with 1 × PBS (Gibco, Grand Island, NY, USA) and permeabilized with 0.1% Triton X (Sigma Chemical, St. Louis, MO, USA) in 1 × PBS (Gibco, Grand Island, NY, USA) for 5 min and blocked for unspecific binding with 0.2% BSA (Merck, DA, Germany) for 30 min. Next, cells were incubated with phalloidin-rhodamine solution (1:100) (Sigma Chemical, St. Louis, MO, USA) in 1 × PBS (Gibco, Grand Island, NY, USA) for 30 min, washed in 1 × PBS (Gibco, Grand Island, NY, USA) three times and added 50% glycerol in 1 × PBS (Gibco, Grand Island, NY, USA). Cell morphology and filopodia were visualized and captured using a fluorescence microscopy (Nikon ECLIPSE Ts2, Tokyo, Japan). Relative number of filopodia/cell was determined the number of filopodia/cell of the artocarpin-treated cells dividing by control cells.

### 4.10. Western Blot Analysis

After artrocarpin treatments, A549, H460, and BEAS-2B cells were lysed with radioimmunoprecipitation assay (RIPA) lysis buffer. The buffer was smented with a protease inhibitor cocktail (Roche Molecular Biochemical). The lysates were collected, and the protein contents were determined by a BCA protein assay kit (Pierce Biotechnology, Rockford, IL, USA). Proteins were subjected to SDS-PAGE, transferred to PVDF membrane (Bio-Rad Laboratories Inc., CA, USA). The membrane was blocked with TBST-T containing 5% (*w*/*v*) non-fat dry milk (Merck, DA, Germany) for 30 min. The membranes were washed with TBST (3 times), incubated with a specific primary antibody at 4 °C overnight. The primary antibodies against interested proteins including Snail, Slug, N-cadherin, E-cadherin, Vimentin, Cav-1, mTOR, p-mTOR, Cdc42, FAK, p-FAK, β-actin, Integrin β1, Integrin β3, Integrin β5, Integrin αv, Integrin α5, Akt, and p-Akt (Cell Signaling, Danvers, MA, USA) were added. After washed with TBS-T, the membranes were incubated with a corresponding secondary antibody (Cell Signaling, Danvers, MA, USA). The protein expression levels were visualized and detected by enhanced chemiluminescence system using Super signal West Pico (Pierce, Rockford, IL, USA) and quantified using ImageJ software (NIH, Bethesda, MD, USA).

### 4.11. RNA Isolation, Reverse Transcription and Quantitative Real-Time PCR (qRT-PCR)

A549 and H460 cells were treated with artocarpin for 24 h. After treatment, the treated cells were collected, and total RNA were extracted using the RNeasy Mini kit according to the protocol of the manufacturer (Qiagen GmbH, Hilden, Germany). RNA was reverse transcribed using a kit from Qiagen (QIAGEN Inc., Maryland, USA), then cDNA was diluted with distilled RNAse/DNase free water. 5 μL of cDNA (20 ng) was used per reaction. The thermocycling conditions were as follows: 95 °C for 15 min; and 50 cycles at 94 °C for 20 s, 50 °C for 30 s, and 72 °C for 20 s. The primers were as follows: N-cadherin forward primer, 5′-GACCGAGAATCACCAAATGTG-3′, reverse primer, 5′-GCGTTCCTGTTCCACTCATAG-3′; Vimentin forward primer, 5′-ACCCTGCAATCT TTCAGACAG-3′, reverse primer, 5′-GATTCCACTTTGCGTTCA AGG-3′; Snail forward primer, 5′ CTAGCGAGTGGTTCTTCTGC 3′, reverse primer, 5′ GTAGTTAGGCTTC CGATTGGG 3′; Slug forward primer, 5′-AGCATTTCAACGCCTCCA-3′, reverse primer, 5′-GGATCTCTGGTTGTGGTATGAC-3′; β-actin forward primer, 5′-AGAGCTACGAG CTGCCTGAC-3′ and reverse primer, 5′-AGCACTGTGTTGGCGTACAG-3′. The expression level of each gene of interest was normalized to the β-actin. All samples were performed in triplicate and the data were calculated using the ΔΔ_Ct_ method.

### 4.12. Immunofluorescence Assay

After treatment, the single spheroids were fixed with 4% paraformaldehyde in 1 × PBS (Gibco, Grand Island, NY, USA) at room temperature in the dark for 20 min and permeabilized with 0.1% Triton-X in 1 × PBS (Gibco, Grand Island, NY, USA) for 15 min. Nonspecific signals were blocked with 3% bovine serum albumin (BSA) for 30 min. The spheroids were washed with 1 × PBS (Gibco, Grand Island, NY, USA) and incubated with anti-CD44 and anti-CD133 antibody at 4 °C for overnight. Spheroids were washed and incubated with Alexa Fluor 488 (Invitrogen) or Alexa Fluor 594 (Invitrogen) conjugated secondary antibody at room temperature in the dark for 1 h. After incubation, spheroids were washed with 1 × PBS (Gibco, Grand Island, NY, USA) and co-stained with Hoechst 33342 (Sigma, St. Louis, MO, USA) for 10 min, then visualized and imaged using fluorescence microscope (Nikon ECLIPSE Ts2, Tokyo, Japan) and the analysis was performed using ImageJ software.

### 4.13. Computational FAK Modelling and Molecular Docking

The binding affinity of artocarpin to important FAK protein and regulators crucial for EMT in human cancer cells was determined using molecular docking methods. To prepare for the docking study, the crystal structures of human FAK (PDB ID: 1MP8) [70] was obtained from Protein Data Bank (PDB). The structure of artocarpin was downloaded from PubChem [71] and converted into pdb format using openbabel [72]. Both pdb files were then converted to pdbqt format using AutoDockTools [73]. We then used AutoDock vina [74] to perform the docking calculation of the artocarpin to the ATP binding pocket. For the molecular dynamics (MD) simulations, the missing amino acid residues of 1MP8 were completed using the Swiss- PdbViewer [75]. We used Avogadro [76] to add hydrogens to artocarpin and used ACPYPE-AnteChamber [77] to generate the mol2 and topology files. We applied the general AMBER force field (GAFF) [78] for the ligand and the AMBER ff14SB force field for the protein [79]. The system was then solvated using the TIP3P water model [80]. The Na^+^ and Cl^-^ ions were added to neutralize the system. The steepest descent was used for energy minimization. We used V-rescale [81] for temperature coupling with coupling constant of 0.1 ps. Th electrostatic and van der Waals interactions were based on the Particle Mesh Ewald (PME) algorithm [82]. The short-range van der Waals (rvdw) electrostatic (rcoulomb) cutoffs and neighbor list (rlist) were set to 12 angstroms. The LINCS algorithm was used to constrain all bond lengths [83]. The time step was set to 0.002 ps. The complex was equilibrated in NVT and then NPT ensembles, each with 100 ps. The molecular dynamics (MD) simulation based on GROMACS 2020.4 was then carried out for 300 ns [84,85]. The heavy atom of artocarpin was then measured for the root mean square deviation (RMSD). The binding free energies were calculated based on MM/GBSA method [86] via the gmx_MMPBSA program [87]. We then evaluated the per-residue decomposition to identify the key amino acids for ligand recognition. The PyMOL molecular graphics program (Schrödinger, Inc.) was used for general structural representation of the FERM domain and publication-quality images were made using the ray-trace command.

### 4.14. Statistical Analysis

All data are presented as the mean ± S.D., derived from at least three independent experiments. GraphPad Prism version 8 (GraphPad Software, San Diego, CA, USA) was used to analyze all data. Statistical analysis was performed by one-way analysis of variance and Turkey’s post hoc test at a significance levels of *p* < 0.05.

## 5. Conclusions

In conclusion, we showed for the first time the novel information that artocarpin suppresses cell migration and survival in anchorage-independent growth conditions with a detailed mode of action of EMT suppression and the decrease of integrins αv and β3, which consequently suppresses downstream migratory proteins. The suppression of the Akt/mTOR signals caused by artocarpin resulted in the inhibition of cancer cell transition to mesenchymal phenotypes. In addition, we found that artocarpin could decrease the level of caveolin-1 and integrins αv and β3, which are known for metastasis potentiation. These data could support the development of artocarpin for anti-metastasis approaches.

## Figures and Tables

**Table 1:** The MM/GBSA (∆_Gbind_) and its energy components (kcal/mol) of the artocarpin-FAK complex.

FAK1-Artocarpin (∆Gbind)	
∆Eele	−35.36 ± 6.11
∆EvdW	−43.15 ± 3.71
∆EMM	−78.51 ± 5.22
∆Gsolv,non-polar	−6.07 ± 0.23
∆Gsolv,polar	40.70 ± 3.72
∆Gtotal	−43.89 ± 3.40
−TDS	14.78
∆Gbind	−29.11

## Data Availability

Data is contained within the article.

## Supplementary Materials

The following are available online at https://www.mdpi.com/article/10.3390/pharmaceutics13040554/s1. Figure S1: Effect of artocarpin on organoids: A549 and H460 cells were treated with artocarpin (10 μM) for 24 h, and allowed for 3, 7, 14, and 21 days to form organoids (A). (B-D) The number of organoids, buds, as well as the relative organoid area were investigated by compared to control. Values represent the mean ± SD. (*n* = 3). * *p* < 0.05 compared with untreated cells. Figure S2: Artocarpin decreased the levels of p-FAK in human lung cancer cells. (A) A549 and (B) H460 cells were treated with non-toxic concentrations of artocarpin for 24 h. Cells were co-stained with anti-p-FAK antibodies, and Hoechst 33342. The expression of p-FAK was examined using immunofluorescence. (C,D) The fluorescence intensity was analyzed by ImageJ software. Values represent the mean ± SD. (*n* = 3). * *p* < 0.05 compared with untreated cells. Video S1: AC and 1MP8.

## Sample Availability

Samples of the compounds are not available from the authors.
