# Supplementary material for: Artocarpin Targets Focal Adhesion Kinase-Dependent Epithelial to Mesenchymal Transition and Suppresses Migratory-Associated Integrins in Lung Cancer Cells
Source: Pharmaceutics. 2021 Apr 14;13(4):554. doi: 10.3390/pharmaceutics13040554 (PMC8071053; doi:10.3390/pharmaceutics13040554)
Supplement: Supplementary file 1 [file pharmaceutics-13-00554-s001.zip › pharmaceutics-1166885 sup for xml.docx]

Supplementary Materials: Artocarpin Targets Focal Adhesion Kinase-Dependent Epithelial to Mesenchymal Transition and Suppresses Migratory-Associated Integrins in Lung Cancer Cells

Nongyao Nonpanya, Kittipong Sanookpan, Nicharat Sriratanasak, Chanida Vinayanuwattikun,
Duangdao Wichadakul, Boonchoo Sritularak and Pithi Chanvorachote

| **Citation:** Nonpanya, N.; Sanookpan, K.; Sriratanasak, N.;  Vinayanuwattikun, C.; Wichadakul, D.; Sritularak, B.; Chanvorachote, P. Artocarpin Targets Focal Adhesion Kinase-Dependent Epithelial to  Mesenchymal Transition and  Suppresses Migratory-Associated  Integrins in Lung Cancer Cells.  *Pharmaceutics* **2021**, *13*, x. https://doi.org/10.3390/xxxxx  Academic Editor: Javier Garcia-Pardo  Received: 17 March 2021  Accepted: 6 April 2021  Published: date  **Publisher’s Note:** MDPI stays  neutral with regard to jurisdictional claims in published maps and  institutional affiliations.  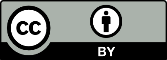  **Copyright:** © 2021 by the authors. Submitted for possible open access publication under the terms and  conditions of the Creative Commons Attribution (CC BY) license (http://creativecommons.org/ licenses/by/4.0/). |
| --- |

**Video S1: AC and 1MP8.** (Available online).


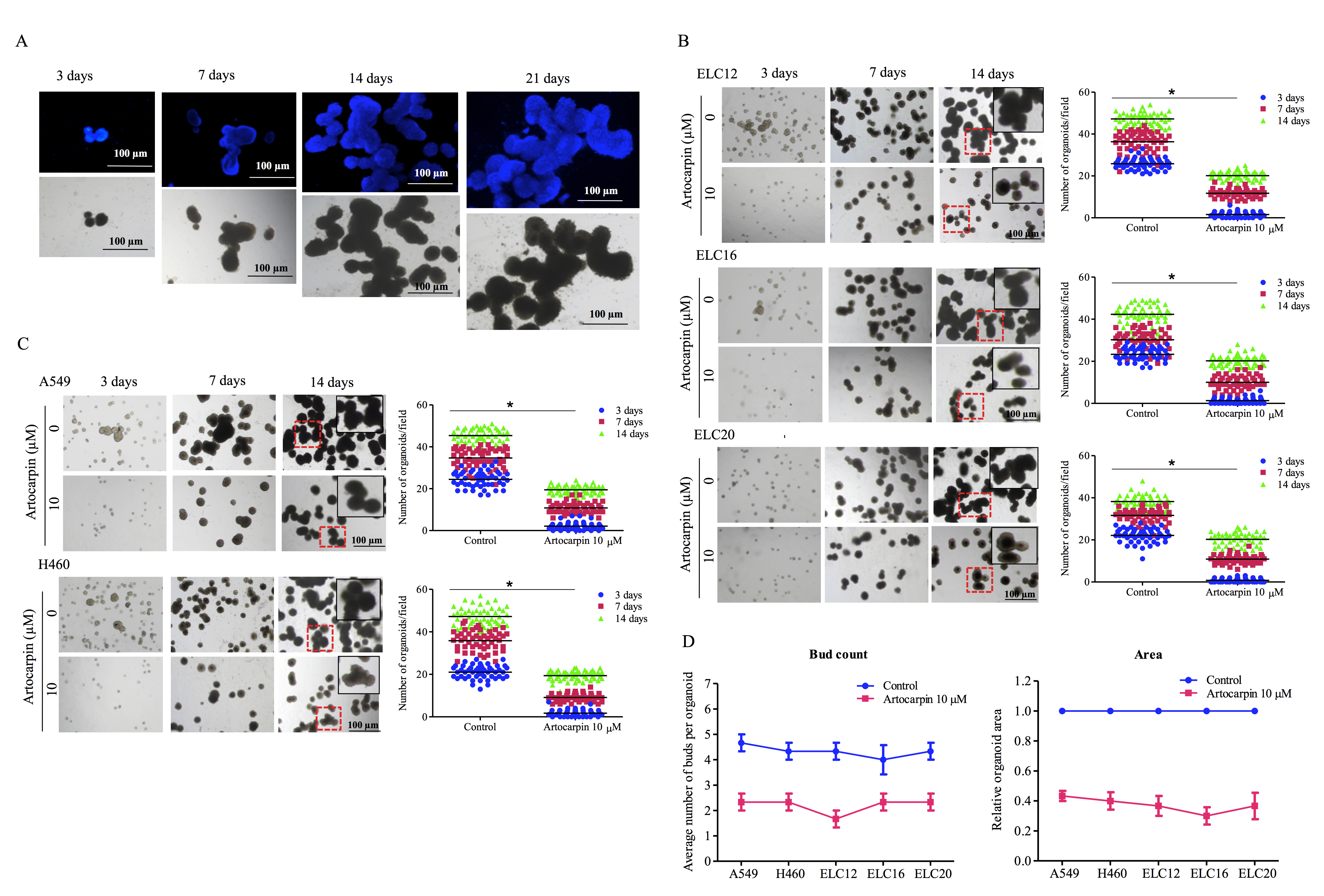


**Figure S1.** Effect of artocarpin on organoids: A549 and H460 cells were treated with artocarpin (10 μM) for 24 h, and allowed for 3, 7, 14, and 21 days to form organoids (**A**). (**B**–**D**) The number of organoids, buds, as well as the relative organoid area were investigated by compared to control. Values represent the mean ± SD. (*n* = 3). * *p* < 0.05 compared with untreated cells.


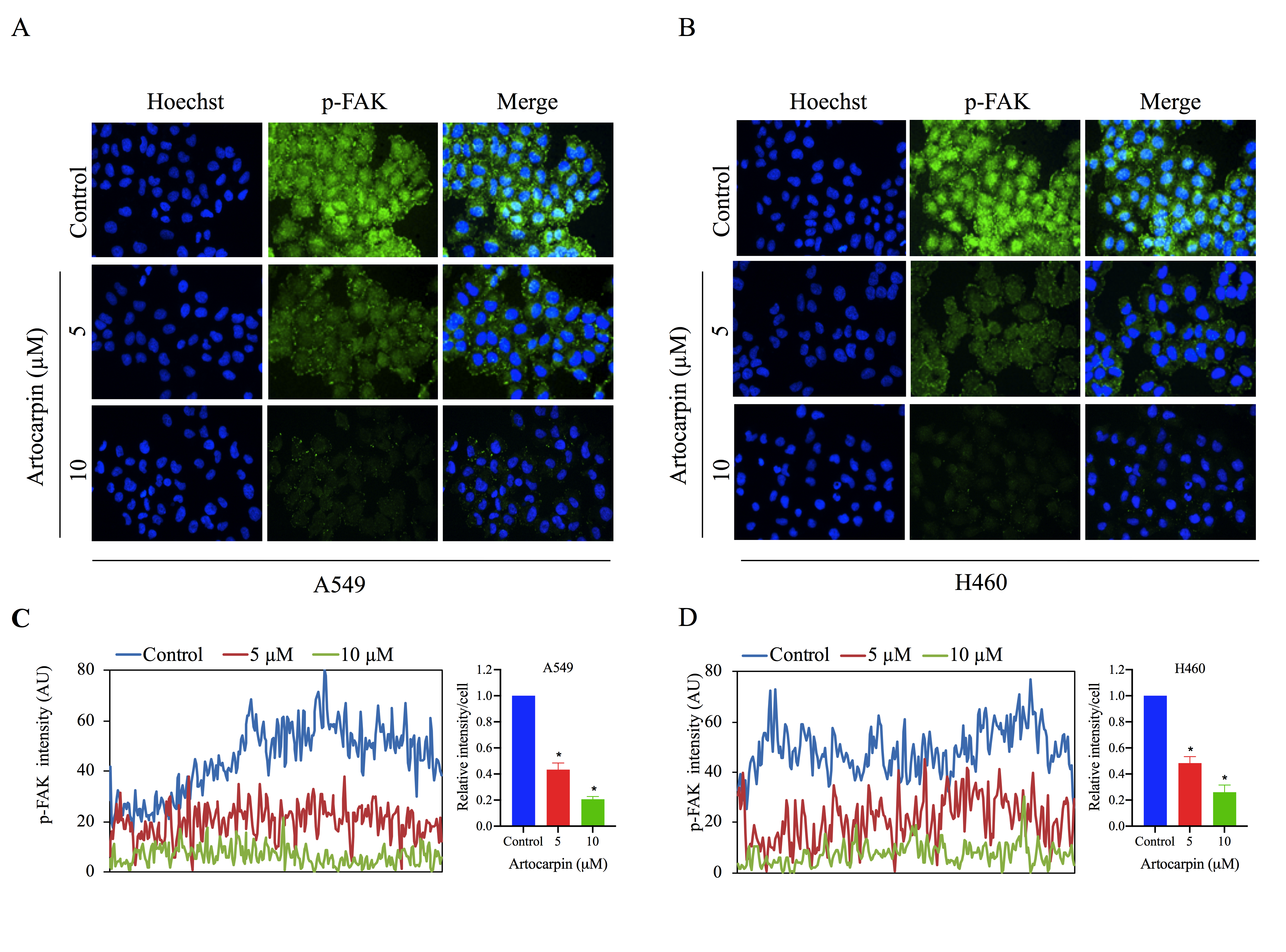


**Figure S2.** Artocarpin decreased the levels of p-FAK in human lung cancer cells. (**A**) A549 and (**B**) H460 cells were treated with non-toxic concentrations of artocarpin for 24 h. Cells were co-stained with anti-p-FAK antibodies, and Hoechst 33342. The expression of p-FAK was examined using immunofluorescence. (**C**,**D**) The fluorescence intensity was analyzed by ImageJ software. Values represent the mean ± SD. (*n* = 3). * *p* < 0.05 compared with untreated cells.
